# The PAR^3^TY Project: Revealing Unique Cancer Experiences and Insights of Teenagers and Young Adults through Patient Engagement, Participation, and Performance

**DOI:** 10.3390/curroncol31100439

**Published:** 2024-10-01

**Authors:** Alice O’Grady, Cheryl A. Heykoop, Will Weigler

**Affiliations:** 1 School of Performance and Cultural Industries, University of Leeds, Leeds LS2 9JT, UK; a.ogrady@leeds.ac.uk; 2 School of Leadership Studies, Royal Roads University, Victoria, BC V9B 5Y2, Canada; 3 Independent Researcher & Theatre Artist, Vancouver, BC V6E 1J3, Canada; will.weigler@gmail.com

**Keywords:** teens and young adults (TYAs), adolescents and young adults (AYAs), cancer, knowledge translation, performance as research, patient activated research, participatory action research, patient-oriented research

## Abstract

Cancer in teenagers and young adults (TYAs) coincides with major life transitions and presents unique psychosocial challenges. Understanding the experiences and needs of TYAs is critical. TYAs want to play an active role in improving cancer for TYAs; however, few opportunities exist for TYAs to do so. Using a tri-partite methodology, an international team collaborated with four TYA co-researchers in this pilot study to explore how performative staging strategies help convey TYA experiences with cancer. Using creative video, TYA co-researchers shared cancer experiences and insights in novel, impactful ways. The process provided intrinsic benefits for co-researchers to connect with other TYAs and creatively share their experiences and perspectives. Furthermore, it provided space for dialogue between TYAs and cancer care allies where TYAs could convey the nuances of their cancer experiences and how cancer care could be improved. This tri-partite methodology can support TYAs to actively engage in a process of connection, reflection, creation, and dissemination to improve cancer experiences for TYAs.

## 1. Introduction

Each year in the UK, approximately 2600 teenagers and young adults (TYAs) are diagnosed with cancer [1]. Globally, the term TYA is often used synonymously with adolescents and young adults (AYAs); however, each country and professional group defines AYA or TYA differently. For example, in Canada, the age range is 15–39; and in the UK, clinical service provision focuses on patients aged 15–24, whereas 15–39 is used to shape the national research agenda [2]. In this article, we use the term TYA as this research was conducted in the UK. Regardless of the term used, evidence indicates that the unique needs of young people are largely unmet by cancer care systems [2,3,4]. Cancer in TYAs often coincides with major life transitions such as post-secondary education or employment, independent living, marriage and partnerships, parenthood or caring for ageing relatives [4], and significantly impacts their life course. The new onset of serious illness also presents unique medical and psychosocial challenges for TYAs including the fear of early death [4], infertility [5], social and financial disruptions [6], and fear for the future [4,7]. It is well-established that TYAs require life-stage appropriate cancer care distinct from children and older adults.

Over the last two decades, cancer care research and practice for TYAs has gained momentum globally and nationally [4,8]. The American Society of Clinical Oncology now recommends that TYAs have specific cancer care and support programs, and, in the UK and Australia, the provision of TYA specific cancer care is standard practice [9]. Furthermore, cancer charities, such as the Teenage Cancer Trust, offer targeted support and programming for TYAs. However, there is more work to be done to improve TYA cancer care and support including the active engagement of TYAs. Research indicates that TYAs have a clear view of how their care can be improved and want to play a more active role in developing approaches that reflect their specific needs [10,11]; however, few opportunities exist for TYAs to influence cancer care policy and practice (ibid). Among the few opportunities, participatory performance arts have been effectively used to (a) make research findings more accessible for patients, families, and healthcare professionals [12] and (b) improve the quality of life of cancer patients [13]. To further address this gap, we created the PAR^3^TY project, the acronym standing for patient activated research, participatory action research, and performance as research (PAR^3^) for Teens and Young Adults.

Conceived as a pilot study, the PAR^3^TY project created an opportunity for a small group of TYAs, based in the UK, to engage in a participatory, arts-based research process to share their lived experiences and insights about TYA cancer care. The ultimate project goal was to use performance as a vehicle to explore and communicate these insights with cancer care allies—family, policymakers, healthcare professionals, organisations, researchers, and supporters—who provide wrap around support during treatment and beyond. The young people involved acted as co-researchers within the project and formed a core part of the research team, playing an active role in the investigation, dissemination, and evaluation.

In this article, we—three international researchers with different disciplinary backgrounds and our own lived experiences with cancer—provide an account of the tri-partite methodology we undertook and how the project was shaped by the ongoing pandemic. We pay particular attention to how a bespoke performance language was introduced, developed, and applied in order to articulate the lived experiences of TYAs living with cancer. We share the key findings and lessons learned emerging from the study, the study limitations, and then conclude by discussing implications for future research and practice.

## 2. Materials and Methods

Our research aim of this pilot study was to develop a methodology that promoted the active engagement of TYAs in a collaborative investigation of TYA experiences with cancer and supported patient agency and influence within the realm of cancer care. Furthermore, we aimed to create a shared environment, mediated by performance, for meaningful dialogue between TYAs and cancer care allies to inform and shape TYA cancer care and support policy and practice. 

For this project, we developed and applied a tri-partite methodology, weaving together three distinct yet complementary, qualitative research methodologies: participatory action research, patient activated research, and performance as research. Below, we explore these methodologies and offer a rationale as to why we undertook this integrated approach. We begin by exploring participatory action research and patient activated research, and then present our orientation of performance as research that integrates principles from the two previously presented methodologies.

### 2.1. Participatory Action Research

Participatory action research refers to an iterative research process conducted with people, rather than on people [14] to explore a situation or action [15] and move towards and/or create social change [14]. Fundamentally, participatory action research is a co-learning process that values people as experts and agents of change in their lives and is action-oriented, aimed towards creating change for people affected [16,17,18]. Furthermore, within participatory action research, arts-based research is recognised for its effectiveness in engaging productively with participants and to generate research findings [19,20,21], and participatory action processes are increasingly considered to be powerful tools to help mitigate power imbalances [16,17] and meaningfully engage underrepresented populations in research [18]. 

### 2.2. Patient Activated Research

Aligned with the principles and ethos of participatory action research, patient activated research (also known as patient-oriented research) refers to research that engages patients as partners, focuses on patient-identified priorities, improves patient outcomes, and applies knowledge generated to improve healthcare systems and practices [22,23]. The key differentiators between participatory action research and patient activated research is that the latter focuses solely on patient engagement and not participants more generally, and unlike participatory action research, action is not always an explicit part of the patient activated research process. Patient activated research builds from the understanding that patient engagement in research can improve research quality [24], knowledge translation efforts [24], informed health decision-making [25,26], and patient-reported outcome measures [26,27]. Patient activated research was a particularly critical methodological orientation to this pilot research as it is increasingly a known methodology in healthcare and supports less traditional forms of research such as arts-based research and performance as research. 

### 2.3. Performance as Research

Performance as research refers to a field of research in which ‘practitioner-scholars draw on rich theatrical and performance traditions to articulate the ways aesthetic interpretation can be understood as a form of academic inquiry’ [28]. Within the discipline, one finds a range of approaches to effectively weave the evocative impact of the arts into what scholars would consider to be ‘legitimate’ research [29]. In their review of variations in performance as research, Prendergast and Belliveau [30] included approaches such as verbatim theatre [31], performance/performed ethnography [32], performed research [33], documentary theatre [34], performative inquiry [35,36], and ethnotheatre/ethnodrama [37,38]. Essentially, all of these variations of performance as research rely on aesthetic means to gather and interpret data solicited from research subjects, which artist-researcher(s) use to promote a more deeply felt understanding and broad dissemination of the findings. Within performance as research, there have been several notable instances where performance has been used as a vehicle to share TYA cancer experiences (see [39,40,41,42,43]); however, these instances of performance as research have not necessarily integrated the principles of participatory action research and patient activated research, as conducted in this pilot study.

Drawing from participatory action research and patient activated research, we engaged TYAs as co-researchers in this pilot study, stepping away from the binary distinction between research subjects who provide the data, and artist-researchers who interpret it and enliven the findings. Through a process intended to privilege their agency, the co-researchers analysed qualitative data drawn from their own experiences using participatory thematic analysis [43,44,45] and determined their own research questions. Then, using performative language as the research method itself, they tested different ‘staging strategies’ to collectively identify performative means that would illuminate their findings in nuanced and complex ways. In an aspiration to “make the undiscussable, discussable” [46], our conception of performance as research in the pilot enabled the co-researchers to ground the explorations, analyses, and interpretations of their findings in physical, aesthetic, and theatrical modalities rather than in expository language and were not simply testimonials or stories.

A significant aspect of our application of performance as research involved relying on the performance equivalent of etudes or studies in drawing or musical composition that seek to capture the fragmentary essence of a specific element of experience without the need for the traditional format of an entire play with characters and a narrative arc. When performed (or screened, in the case of short videos), the audience, moved by the aesthetic impact of the etude, is invited into the research space and can engage in a conversation with the researchers/performers and the stakeholders/audiences about productive actions that could be taken to advocate and support change for TYAs navigating cancer.

### 2.4. Weaving the PARs Together

Drawing upon our distinct interpretations and understandings of the research methodologies described above, we created an integrated, tri-partite methodology (PAR^3^) for this pilot project. Our PAR^3^ methodology sought to value people as experts and agents of change in their lives [14,46,47]; adopt a co-learning process that can help mitigate, or move beyond, power imbalances [16,17,48]; employ storytelling and theatre as methods of knowledge translation and learning [24,49,50]; build the capacity of patients to engage in research [22]; support relevant, effective knowledge translation by engaging patients and research users in knowledge synthesis, dissemination, and application [22,49,50]; and transform healthcare systems and practices to improve patient outcomes [22,23,51,52]. Below, we discuss how we applied the PAR^3^ methodology within our pilot study.

### 2.5. Study Conduct

#### 2.5.1. Study Design

Drawing upon our individual disciplinary perspectives and research experiences, we developed a collaborative, co-created approach focused explicitly on TYA cancer care transformation that placed the young people as co-researchers at the centre of the research and supported young people to discover ways to express their insights through vivid, evocative, and powerful theatrical form. Specifically, building on the existing work of Heykoop in participatory action research (see: [53,54]) and young adult cancer care transformation, we grounded our approach on the latest research on TYA cancer care and support. Drawing upon O’Grady’s applied theatre work in risk, participation, and performance (see: [55]), we integrated ethical storytelling and edge play. By adopting the theatre-based staging strategy approach developed by Weigler in The Alchemy of Astonishment [50], we engaged young people in a process that mobilised performance as the method for sharing experiences, processing them, and knowledge dissemination. 

Originally, the pilot project was scheduled to take place as an intensive one-week workshop and devising period in April 2021 based at the University of Leeds. The intention was to spend time with TYA co-researchers as an ensemble to share experiences, experiment with staging strategies, and co-create an immersive performance piece for a live audience made up of health professionals, family members, and other cancer care allies. However, the ongoing restrictions caused by COVID-19 prevented us from meeting face-to-face. We then reworked the pilot project for online delivery, gained full ethical approval, and received a project extension to accommodate the pivot to online.

#### 2.5.2. Recruitment

The project sought to engage TYA co-researchers who had been diagnosed with cancer in the UK between the ages of 16 and 24, and who were over the age of 18 at the time of the study. Participants were required to have been out of active treatment for over 12 months, as we wanted to ensure that the TYAs involved felt well enough physically and emotionally to participate and had had some time to reflect upon their experiences with cancer (including diagnosis and treatment). 

Despite our best efforts, recruiting TYA co-researchers proved challenging in the wake of COVID-19 and lockdowns. Many cancer care support groups had ceased meeting in person, and in some instances, cancer centres had lost contact with TYAs post-active treatment. Furthermore, many young people were experiencing Zoom fatigue and were not particularly interested in joining an online study. To assist with recruitment, we worked closely with local cancer care allies in the Yorkshire region and charities, such as Clic Sargent and Teenage Cancer Trust, to reach out to TYAs and advertise the opportunity via social media. Although approximately 15 young people expressed initial interest, four TYAs committed to the project and engaged fully as co-researchers from conception to dissemination. We did not ask the TYAs involved in the study to disclose their identities as this was not the focus of this research pilot, rather it was on the piloting of the methodology. As such, we did not gather demographic information. In future, we would suggest that this methodology be piloted with TYAs with divers intersectionalities and identities to further explore relevance and suitability. 

#### 2.5.3. Preparing for Co-Researcher Engagement

##### Crafting the Invitation for Engagement

Before the pilot began, we spent considerable time crafting the invitation to participate to set the project tone and signal our commitment to co-created, participatory research. Specifically, we emphasised that the pilot was an opportunity for participants to: (1) take the lead in deciding what they wanted to say about TYA cancer and how they wanted to share it; (2) contribute their own knowledge while learning from one another and collectively consider how to promote positive change in TYA cancer care and support; and (3) learn new techniques to translate ideas and feelings into theatre performances designed to inspire change in the cancer care system. Given the participatory nature of the research, we also reinforced that TYAs would receive a GBP 200 honorarium to help compensate them for their time involved.

##### Preparing for Engagement

As a team, we were deeply committed to creating the conditions for relationship building, story sharing, creative engagement, and co-generation. We met weekly for several months to prepare for engagement with the TYAs and crafted the arc of the six sessions. Prior to our first meeting with the TYAs, we also sent a ‘creativity kit’ to the co-researchers through the post. This kit, intended to support creative engagement in the project, included a journal, a pen, colouring pencils, modelling clay, Post-it notes, tissues, and a deck of Will’s Alchemy of Astonishment [50] staging strategy cards, which prompt the learning of theatrical vocabulary and its implementation into a scene or performance. 

#### 2.5.4. Implementing the Pilot

The redesigned online pilot project took place over Zoom between April and June 2021. The 12-week period involved six fortnightly sessions with the research team and co-researchers. During the off weeks, the co-researchers were encouraged to continue to reflect, distil, and process their experiences and the material emerging from each workshop session, while the project team met to adapt and re-structure the enquiry in progress. With the group’s permission, a WhatsApp group was established to also facilitate communication between sessions and send prompts between the fortnightly workshops. 

To facilitate the online sessions, we used group activities and exercises common to applied theatre practice to help facilitate open collaboration and expression. Each session, we spent time to build relationships, trust, and shared ways of working; share creative prompts and techniques; and exchange stories about our experiences with cancer. The first two sessions focused primarily on building trust and story sharing, the third session explored key themes about TYA cancer, sessions 4 and 5 focused on crafting and devising, and session 6 offered an opportunity to share the project outputs with friends, family, and supporters. In between sessions, the co-researchers were given a creative prompt to consider and were invited to write in their journals and share thoughts on what was particularly difficult to explain or verbalise to someone who had not had first-hand experience of cancer themselves—family, friends, and/or healthcare advocates. 

Throughout the 12-week-period, the group was encouraged to use the material in their creativity kits (including the staging strategy cards) to express their ideas. It was here that the research adopted a different approach to many of the other works in this field (see performance as research and Lived Experiences of TYAs section above). Rather than encouraging the group to convey their feelings and experiences in a particular way, we invited them to experiment with the art-making materials and staging strategy card techniques to convey aspects of TYA cancer care and support that were difficult to explain and/or were unexplainable. Each TYA was then invited to design and create a short video that integrated the staging strategies in an intentional and crafted way, where the co-researchers were able to rely on the way of staging ideas to convey experience and insights, rather than relying only on words. 

To offer an illustrative example of how the staging strategies were particularly helpful, we share how one co-researcher drew upon two staging strategy cards to craft her performative video about her complex relationship with cancer. To help convey her insights, she drew from the cards Withholding and Parallel Tracks with a Twist. The first staging technique refers to withholding visual clues from the viewer—in this case, the co-researcher chose to show only a young woman’s hands as she wrote a letter. The second staging technique involves laying down a series of clues that lead the viewer to believe that they know what is happening in the story—in this case, that the young woman was writing a letter to a former romantic partner. The clues are laid down like railway tracks, but at the end of the story, a new piece of information makes it clear to the viewer that there are two parallel sets of tracks, and that the story has been about something else altogether—in this case, the story is actually about a young woman writing a letter to cancer. That unexpected revelation carried an impact for the viewers. By integrating techniques like these, the co-researcher was able to rely on the staging of her video to convey her experience, rather than relying only on the words to explain this complex aspect of her challenges post-treatment as a TYA. 

The pilot project concluded with a series of video showcases, where the four co-researchers shared their powerful videos (see Supplementary Materials) about TYA cancer care and engaged in conversation with an invited, online audience of cancer care allies from the UK and Canada. Interviews were conducted with the TYA co-researchers to learn more about their experiences and insights emerging from the pilot, and the videos continue to be shared with researchers and health professionals interested in TYA cancer care. Interview recordings were transcribed along with hand-written notes from the six sessions, and thematic analysis was conducted using Braun and Clarke’s six step process to generate codes and themes [56]. Key themes were then discussed with the TYAs involved in the study to further verify the study findings.

### 2.6. Study Limitations

As noted above, this pilot study involved four TYA participants, which may limit the generalisability of the findings. However, given that this article focused on the research process and not on the experiences of TYAs navigating cancer care and support, the lessons learned are relevant for future research studies. This pilot study engaged TYAs who were 12 months post-treatment, and it is unclear if and how it would be suitable for TYAs in active treatment. Furthermore, given that we did not collect the demographic information of the participants, future research could explore the suitability and applicability of this methodology with TYAs with diverse intersectionalities and identities.

## 3. Results

Although the PAR^3^TY project was a pilot study and involved only four TYA participants, it produced significant findings about both the research process and outcomes, which we offer below for consideration and have integrated into subsequent research projects with TYAs and cancer care allies. It is important to note that we are not sharing the findings about the TYA cancer experiences that emerged through the study, rather, our focus was on the on learnings related to the research process itself and their potential to influence change in TYA cancer care and support. 

### 3.1. Video Etudes Offer Effective Way to Share about the Nuances of TYA Cancer

At the showcase events, cancer care allies—friends, supporters, healthcare providers, decision-makers, researchers, and organisations—used phrases such as “very emotive”, “powerful”, “simply crafted and very thought provoking”, “very clever”, and “deeply moving” to express and share their reactions to the videos. Cancer care allies spoke to how the videos supported TYAs to share their lived experiences in ways that offered an alternative to the testimonials or common tropes that accompanied cancer narratives and instead focused on capturing the essence of the AYA cancer care experiences. For example, as one family member shared: “I loved that the project allowed the team to be really creative, using their own voices to produce something very different from ‘normal’ cancer stories”. Others noted how the videos helped capture the nuances and complexities of TYA cancer care. One healthcare provider noted how they were “struck by how they [the videos] turned assumptions on their head”, and another participant from a community organisation pointed out that the video depicting the writing of a love letter “challenged the assumptions that cancer is the cause of negative feelings only but is in fact more complex than that, like a relationship”. Cancer care allies also reflected upon the value of the videos to be a “powerful educator” to “bring people in”, “build understanding and empathy”, and promote active dialogue between cancer care allies and TYAs to improve, and even transform, cancer care. In essence, these videos are powerful tools to facilitate knowledge translation. To date, these videos have been shared with TYAs in Canada to create a series of videos about TYA cancer care and support experiences, and have been shard with cancer care allies in the UK and Canada to promote conversations about how TYA cancer care and support systems can be improved.

### 3.2. Process Positions TYAs as Experts and Offers Opportunities to Exercise Agency

Aligned with participatory action research, the videos (and the research methodology itself) position TYAs as experts of their own cancer experiences and provide TYAs with opportunities to self-determine what and how they wish to share their unique experiences and perspectives beyond narrating their personal story. Furthermore, the approach offers TYAs the opportunity to exercise agency, determining whether they want to deliver or perform the source material or have it delivered by someone else. For example, in preparing her piece, one TYA co-researcher chose to write a film script, complete with camera angles and editing notes, as she felt that she was not emotionally prepared to perform it. Instead, we found an aspiring actor to perform the piece. The fact the co-researcher did not perform the piece herself did not diminish the effectiveness of the work. Rather, the piece stood on its own merit, separate from the individual who had conceived it, and was received by the audience as a powerful revelation about the ambivalent feelings that can accompany the end of active treatment for TYAs. This instance was a testament to the power of the research methodology to capture TYA experiences that go beyond the individual and empower TYAs to share their experiences in creative and impactful ways and spark conversations about creating change in TYA cancer care and support.

### 3.3. Process Offered Opportunities to Connect and Reflect with Other TYAs

The TYAs involved in this pilot study spoke not only to the power of the research outputs—the videos—but also about the beneficial nature of the research process itself. In particular, the TYA co-researchers spoke to how the project was initially of interest to them because it offered an opportunity to connect with others who had experienced cancer as TYAs. As one participant shared, “I was hoping to get some friends and some people to talk to about cancer because I don’t like talking to my friends that don’t have cancer backgrounds. I feel like they don’t understand the same way that people who have had cancer do”. While common for TYAs to undergo treatment on a ward with people of a similar age in the UK, opportunities for post-treatment reflection seem to be rarer, as contact with other TYAs often ceases when treatment ends. The TYA co-researchers also spoke about the value of connecting post-treatment and having time to process and reflect on how cancer impacts TYAs. As one co-researcher shared, “the research process reminded me of the complexities of being someone who’s going through cancer, because none of us want to be defined by our cancer, but we don’t want [it] to be forgotten”. Another TYA co-researcher shared how “comforting” it was to engage in a process where everyone looked normal, but “there is a whole other side to people that nobody really knows”, and through the research process, TYAs could share those hidden aspects with others who “get it”. This finding about the value of connecting with other TYAs is consistent with other research studies, reinforcing the importance of community when navigating a cancer care system that often is not designed for TYAs. 

### 3.4. TYAs Experienced a Sense of Value through the Research Process

All of the TYA co-researchers spoke about how they felt seen, heard, and valued through the research process, and felt that they could speak whatever was on their mind. One TYA shared that they felt welcome to “feel a lot of things” and “speak whatever is truthful for you, no matter how controversial”. The TYA co-researchers also shared how this research was refreshingly different from other studies. As one TYA noted, the research process “felt a lot more comfortable and natural”, and another TYA shared that it “didn’t feel like there were researchers and participants. I felt like we were on level ground, and it was just all of us trying to make something that would explain our own experiences with cancer, which was really, really nice, because I’ve been in other studies where it really felt like they were researchers, they wanted to find out my experience, and then they were done”. It is clear that TYAs want opportunities to share their expertise and insights [10,11], and this research pilot supported TYAs to convey their experiences and advocate for changes in cancer care systems.

### 3.5. Process Offered New, Creative Ways for Expression

All of the TYAs were excited to participate in the research and learn new, creative ways to express their experiences with cancer, and for half of the TYA co-researchers, working with performance was entirely new. The TYA co-researchers all mentioned feeling nervous and excited about the project, but they entered the work with an open mind and felt that, despite some uncertainty about the outputs they were required to produce, the project exceeded their expectations. In particular, they all commented on the importance of the “casual but inviting” open research space that created an environment for conversation that was both “natural and dynamic”, and helped them to realise that they “can do more creative things”. This pilot study offers some possibilities for TYAs to be involved in research about TYA cancer care and support in creative, participatory ways that are often not part of the status quo in healthcare research.

### 3.6. Process Offered Opportunities for TYAs to Share and Exercise Influence with Cancer Care Allies

All of the TYA co-researchers acknowledged that significant learning occurred between the third and fifth workshop when they began to shift focus to what they wished cancer care allies could really understand about TYA experience. Specifically, in the third workshop, we themed the emerging stories and insights about TYA cancer, and upon reflecting on the themes, the overarching feeling described by the co-researchers was “not quite anger but frustration” about TYA cancer and how things could change for the better. The frustration was then harnessed to explore ways to share these hard to express aspects of TYA cancer experiences with those responsible for their care and support. Reflecting on sharing the videos with cancer care allies, the TYAs commented on how they were both surprised and validated to learn that the insights shared were new and fresh. As one co-researcher commented:

Playing those videos that we’d seen multiple times before, but then showing it to a new audience and with people who have worked in cancer charities for years and years and years, I realised they saw things that they didn’t know or understand before watching it. It was interesting to see other people receive those feelings. It’s one thing to sit amongst people who have shared [experiences] with you […] and then it’s another thing to go, right now, we need other people to understand.

The video performance offered a ’safe place’ for TYAs to share their insights with cancer care allies, and the facilitated discussion provided an opportunity for the young people and their allies to have deeper conversations about how it felt to be a TYA with cancer. As one co-researcher explained, having the space to voice fewer positive thoughts and feelings provided a “deep sense of validation” to emotions that had not previously been aired. Furthermore, the TYA co-researchers expressed that the video material could offer TYAs with cancer and cancer care allies more nuanced and raw experiences of cancer care and support and could help to see patients as experts in their own experiences with cancer and as people first. It is interesting to note that the participants in this pilot study had been out of treatment for over 12 months, and TYAs in active treatment may not experience the same sense of validation and safety, as it could impact TYA and cancer care ally relationships and treatment. Furthermore, there was general consensus amongst the research participants about their experiences navigating cancer care as a TYA and this research process; hence there was limited disagreement throughout the pilot study.

## 4. Conclusions

### 4.1. PAR^3^ Methodology Has the Potential to Transform Practice through the Meaningful Engagement of TYAs

This pilot project provided an opportunity for us to develop and test our PAR^3^ tri-partite methodology with a small group of four TYAs co-researchers. The creative research process supported the TYA co-researchers to make sense of their lived experiences with cancer with other TYAs and creatively share their insights and perspectives through video using performative staging strategy techniques. Rather than dramatising TYA testimonials or personal stories (which has been carried out by [39,40,41,42] and others), this novel research process helped generate aesthetic insights about cancer for TYAs that used metaphor and imagery to convey experiences about being a TYA that were difficult to articulate in words, and created opportunities for TYAs to exercise agency in determining how they wanted their insights shared and performed. The pilot supported TYAs to play an active role in determining how cancer care and support for TYAs could be strengthened and improved—a role that is often overlooked in TYA research and care [10,11] and reinforced for cancer care allies both the value and importance of TYA perspectives to shape practice. Cancer care allies who participated in this pilot reinforced the value of how these videos or etudes captured TYA experiences navigating cancer care and saw opportunities for these to be shared with cancer care allies in both the UK, Canada, and beyond. It is our hope that moving forward, there are more opportunities for TYAs to share their insights and experiences with cancer care allies and influence change in practice.

### 4.2. Video Etudes and Dialogues Offer Opportunities to Inform and Shape Cancer Care and Support

The videos created through this pilot study are an important knowledge translation output for TYAs and cancer care allies. For TYAs, the videos offer opportunities to share TYA experiences and what one might expect as they navigate cancer care and support as a TYA. For cancer care allies, the videos are a powerful professional development tool to better understand the lived experiences, needs, and priorities of TYAs. Furthermore, the dialogues amongst TYAs and cancer care allies offer opportunities to discuss and explore how best to improve TYA cancer care from the perspectives of TYAs themselves.

### 4.3. Online Delivery Enables Access for TYAs from Different Locations to Engage

Although not part of the original plan, the transition to a fully online research process offered us opportunities to engage TYAs from different locations across the UK, and ultimately made the project more accessible. Participants could join from anywhere, did not need to arrange transport or accommodation, and could balance their involvement with existing commitments. Moving forward, online delivery could support TYAs who are not feeling physically well but wish to engage in research. The functionality of Zoom and WhatsApp supported ongoing relationship building in ways that may not have been possible in a live setting. Furthermore, working over an extended period provided extra time for the reflection, distillation, and processing of material emerging from the sessions, which would likely not have been achievable over a one-week intensive workshop. In future iterations of PAR^3^ methodology, online elements will be integral to our approach to support access and sustained relationships for TYAs.

### 4.4. Future Research

As a pilot study, this project has been integral to evolving the PAR^3^ methodology and subsequent research projects. Specifically, we adapted our approach to engage young adults across Canada diagnosed with cancer between the ages of 15 and 39 in a six-week virtual workshop series to co-create videos that capture the nuances and complexities of cancer care for TYAs. We are also applying the PAR^3^ methodology through a hybrid process consisting of both online and in-person engagements that will culminate in a modular, multi-sensory, immersive theatre performance. Finally, we are considering how to adapt the process to better support the engagement of TYAs with diverse intersectionalities and identities to inform and shape cancer care and support for all TYAs. 

## Data Availability

The data associated with this paper are openly available from the University of Leeds Data Repository: http://doi.org/10.5518/1390.

## Supplementary Materials

The videos can be accessed here: https://vimeo.com/user134119418 (accessed on 25 September 2024).
